# Total Thyroidectomy for Severe Thyrotoxicosis in a Young Woman: A Case Report

**DOI:** 10.7759/cureus.107104

**Published:** 2026-04-15

**Authors:** Hugo E Mora Moreno, Ulises S Sánchez Guevara, Maria G Maciel García, César A Durán Ramírez, Bryan G Vasquez Marta

**Affiliations:** 1 General Surgery, Hospital General Dr. Miguel Silva, Morelia, MEX

**Keywords:** case report, graves’ disease, hyperthyroidism, thyrotoxicosis, total thyroidectomy

## Abstract

Graves’ disease is the most common cause of autoimmune hyperthyroidism and is typically managed with antithyroid medications, radioactive iodine, or surgery. However, a subset of patients develops persistent thyrotoxicosis or severe complications requiring definitive treatment. We report the case of a 23-year-old woman with Graves’ disease and type 1 diabetes mellitus who presented with persistent thyrotoxicosis despite prolonged treatment with methimazole and beta-blockers, with a prior history of diabetic ketoacidosis complicated by thyroid storm. Physical examination revealed a diffuse grade III goiter with clinical features of hyperthyroidism, while laboratory tests showed suppressed thyroid-stimulating hormone and markedly elevated free thyroid hormone levels. Imaging demonstrated diffuse thyroid enlargement with increased vascularity and mild tracheal compression. Following multidisciplinary evaluation, the patient underwent total thyroidectomy with an uneventful postoperative course, and histopathology confirmed diffuse hyperplastic goiter with chronic thyroiditis. This case highlights total thyroidectomy as a safe and effective definitive treatment for refractory thyrotoxicosis, and suggests that early surgical intervention may prevent life-threatening complications, such as thyroid storm, particularly in patients with complex metabolic comorbidities.

## Introduction

Graves' disease is the most common cause of endogenous hyperthyroidism and is characterized by autoimmune stimulation of the thyroid gland that results in excessive production of thyroid hormones and clinical thyrotoxicosis [1]. The disease predominantly affects young adults, particularly women, and may present with a broad spectrum of manifestations including tachycardia, weight loss, tremor, heat intolerance, and neuropsychiatric symptoms [1,2]. In severe cases, uncontrolled hyperthyroidism may progress to life-threatening complications, such as thyroid storm, an endocrine emergency associated with significant morbidity and mortality if not promptly recognized and treated [2].

The management of Graves’ disease involves three principal therapeutic strategies: antithyroid medications, radioactive iodine therapy, and surgery [3]. Antithyroid drugs, particularly methimazole and propylthiouracil, are commonly used as first-line treatment; however, a considerable proportion of patients experience persistent hyperthyroidism, relapse after treatment discontinuation, or adverse reactions that limit long-term medical therapy [3,4]. In these situations, definitive treatment options must be considered.

Total thyroidectomy has emerged as an effective and definitive therapeutic option for selected patients with Graves’ disease [3]. Surgical indications include failure of medical therapy, intolerance or contraindications to antithyroid medications, large goiters with compressive symptoms, suspicion of coexisting thyroid malignancy, and clinical scenarios in which rapid control of hyperthyroidism is required [1,3]. Compared with other treatment modalities, surgery provides immediate control of hormone excess and eliminates the antigenic stimulus that drives the autoimmune process.

Despite its effectiveness, total thyroidectomy remains a technically demanding procedure that carries potential risks, including recurrent laryngeal nerve injury and postoperative hypoparathyroidism. However, these complications can be significantly minimized when surgery is performed by experienced thyroid surgeons within specialized centers [2,4].

In this report, we present the case of a young woman with severe Graves’ disease refractory to prolonged medical therapy who was successfully treated with total thyroidectomy. The coexistence of uncontrolled hyperthyroidism and type 1 diabetes mellitus represents a particularly high-risk metabolic state. Thyrotoxicosis is known to increase insulin resistance, enhance hepatic gluconeogenesis, and accelerate gastrointestinal glucose absorption, thereby worsening glycemic control. In patients with type 1 diabetes mellitus, these effects may precipitate or exacerbate diabetic ketoacidosis, particularly in the setting of severe hyperthyroidism or thyroid storm. This interaction creates a vicious cycle of metabolic instability, in which persistent thyrotoxicosis contributes to recurrent metabolic decompensation. In this context, definitive treatment with thyroidectomy may play a critical role not only in achieving control of hyperthyroidism but also in improving overall metabolic stability and reducing the risk of life-threatening complications.

## Case presentation

A 23-year-old woman with a known diagnosis of Graves' disease presented with persistent symptoms of thyrotoxicosis despite ongoing medical therapy. Her past medical history was significant for type 1 diabetes mellitus diagnosed at the age of 13 years, treated with insulin glargine (20-0-10 IU) and insulin lispro (5-5-5 IU). She reported an allergy to dexamethasone.

Two years before the current admission, the patient presented to the emergency department with palpitations, vomiting, diarrhea, and hallucinations. She was diagnosed with diabetic ketoacidosis complicated by thyroid storm. Since that episode, she had been treated with methimazole 50 mg daily and propranolol 40 mg as needed. Prior to surgery, preoperative optimization included continuation of high-dose antithyroid therapy and beta-blockade. Additionally, Lugol’s iodine solution was administered in the days preceding surgery to reduce thyroid hormone release and gland vascularity. Despite these measures, the patient remained biochemically hyperthyroid, consistent with refractory disease. Given her history of thyroid storm and ongoing metabolic instability, definitive surgical management was pursued in a controlled setting. Despite this therapy, she continued to experience persistent tachycardia that limited her physical activity. Postprandial capillary glucose levels frequently exceeded 300 mg/dL, requiring additional corrective doses of insulin lispro (3-4 IU).

**Figure 1:**
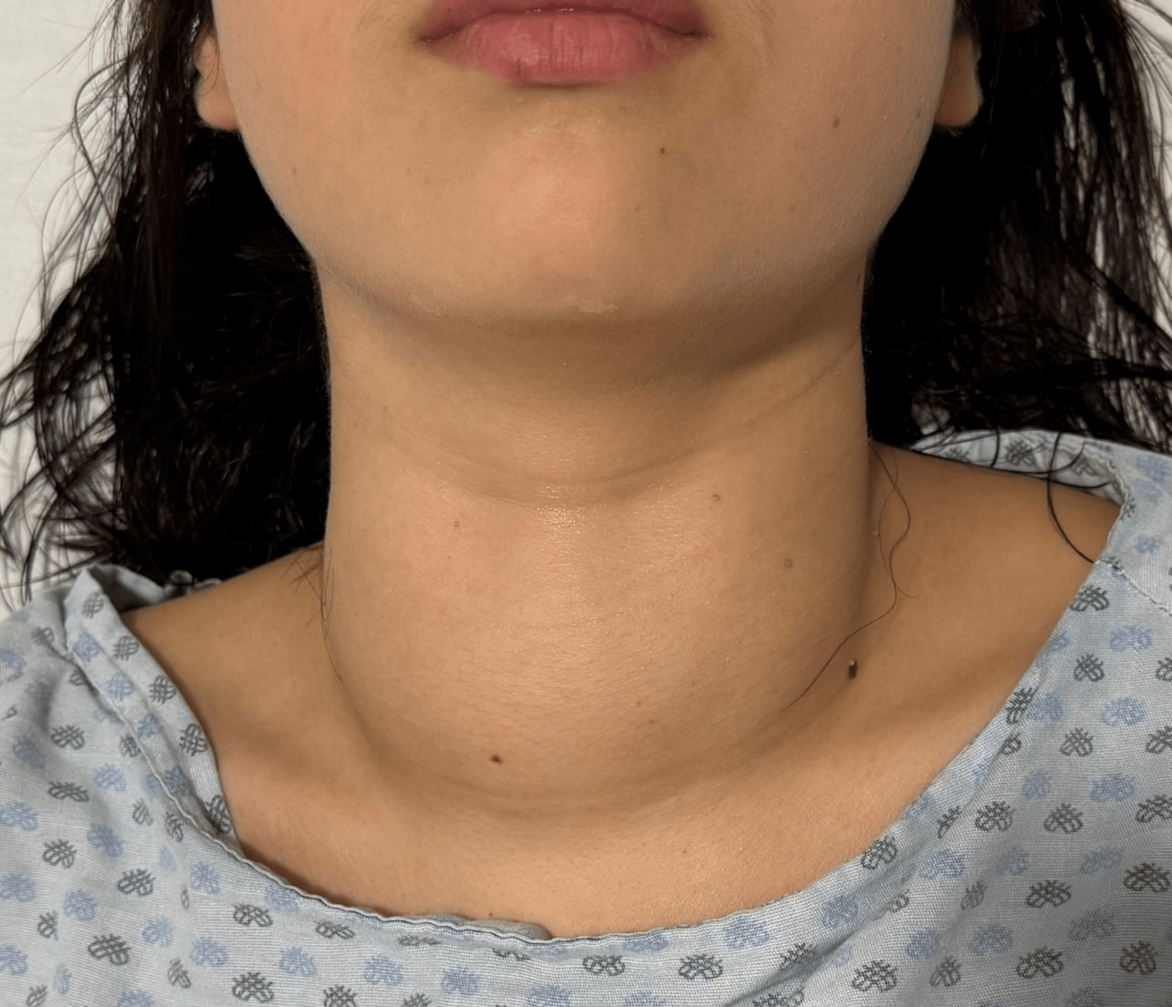
Preoperative photograph showing visible anterior cervical enlargement consistent with diffuse goiter.

On physical examination, the patient was conscious, alert, and oriented, without focal neurological deficits. No clinical signs of ophthalmopathy were observed. Neck examination revealed a grade III diffuse goiter with an estimated thyroid size of approximately 8 × 5 × 3 cm per lobe and an audible bruit over both lobes on auscultation (Figure 1). Cardiovascular examination demonstrated a regular rhythm with a heart rate of 105 beats per minute and no audible murmurs. Pulmonary auscultation was normal, and the abdomen was soft and non-tender. Neurological examination revealed hyperreflexia (+++/+++) and a distal tremor (+++/+++). A 2-cm indurated scar with elevated borders was noted on the left arm corresponding to the previous implant site. Peripheral pulses were palpable, and no peripheral edema was present. 

Preoperative laboratory findings are summarized in Table 1. All other laboratory parameters were within normal limits.

**Table 1:** Preoperative laboratory findings. FT3, free triiodothyronine; FT4, free thyroxine; PTH, parathyroid hormone; TSH, thyroid-stimulating hormone

Parameter	Preoperative	Reference Range
Thyroid-stimulating hormone (TSH)	0.02 mIU/L ↓	0.4-4.0 mIU/L
Free thyroxine (FT4)	4.58 ng/dL ↑	0.8-1.8 ng/dL
Free triiodothyronine (FT3)	123 pg/mL ↑	2.3-4.2 pg/mL
Serum calcium	9.2 mg/dL	8.5-10.5 mg/dL
Parathyroid hormone (PTH)	42 pg/mL	15-65 pg/mL
Serum glucose	270 mg/dL ↑	70-100 mg/dL

**Figure 2:**
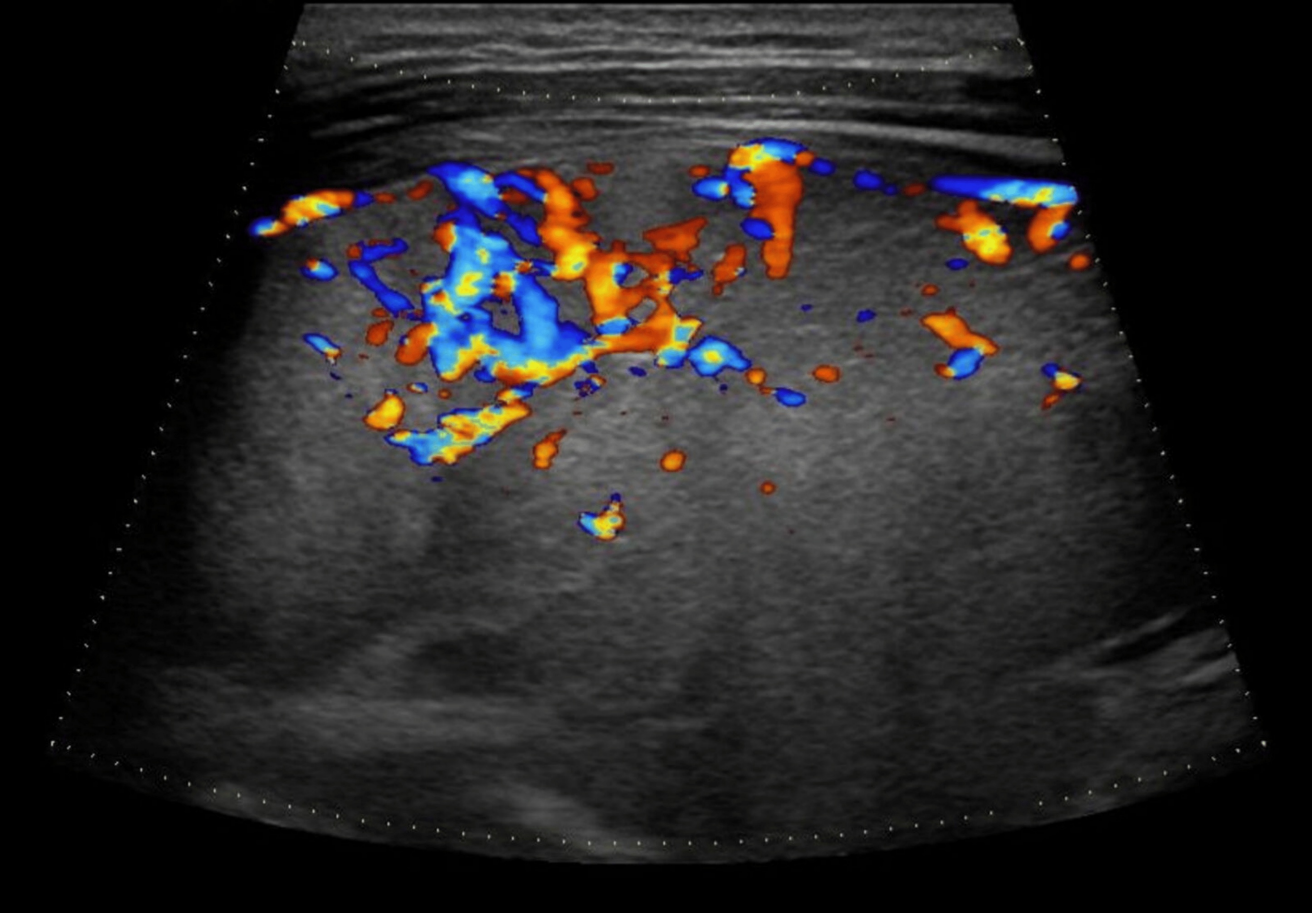
Color Doppler ultrasound demonstrating diffuse hypervascularity of the thyroid gland (“thyroid inferno”) characteristic of Graves’ disease.

Neck ultrasound demonstrated diffuse thyroid enlargement with heterogeneous echotexture. Color Doppler imaging revealed marked hypervascularity throughout the gland, a characteristic finding in Graves’ disease commonly referred to as the “thyroid inferno” pattern (Figure 2). The right thyroid lobe measured 39.6 × 38.3 × 54.3 mm, and the left lobe measured 32.8 × 37.4 × 50.6 mm.

**Figure 3:**
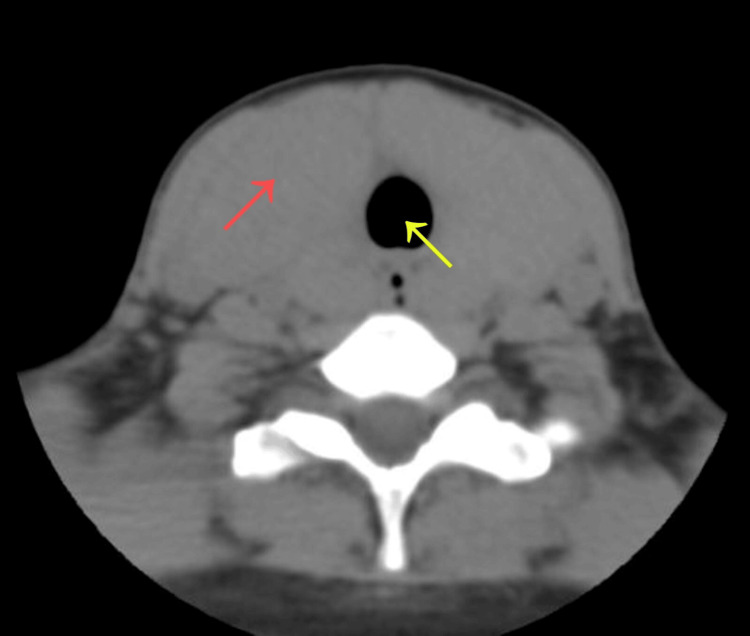
Axial CT scan of the neck showing diffuse enlargement of the thyroid gland (red arrow) causing tracheal compression and anterior displacement (yellow arrow).

**Figure 4:**
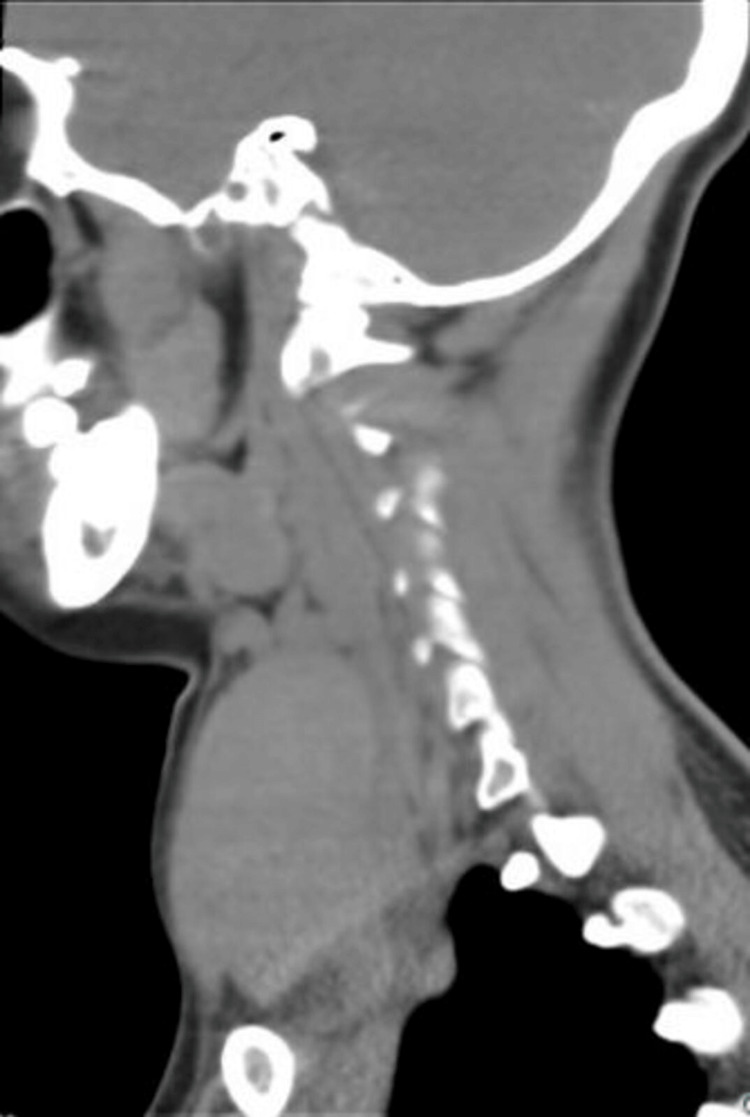
Sagittal CT reconstruction demonstrating diffuse enlargement of the thyroid gland.

Computed tomography of the neck confirmed diffuse enlargement of the thyroid gland with mild tracheal compression and anterior displacement of the airway (Figure 3). Key findings are highlighted with arrows to facilitate visualization. Sagittal reconstruction further demonstrated diffuse enlargement of the thyroid gland (Figure 4). The right lobe measured 4.4 × 5.2 × 6.0 cm, and the left lobe measured 4.2 × 5.1 × 6.9 cm.

After multidisciplinary evaluation by the endocrinology and general surgery teams, the patient was considered a candidate for definitive surgical management due to persistent thyrotoxicosis refractory to medical therapy.

**Figure 5:**
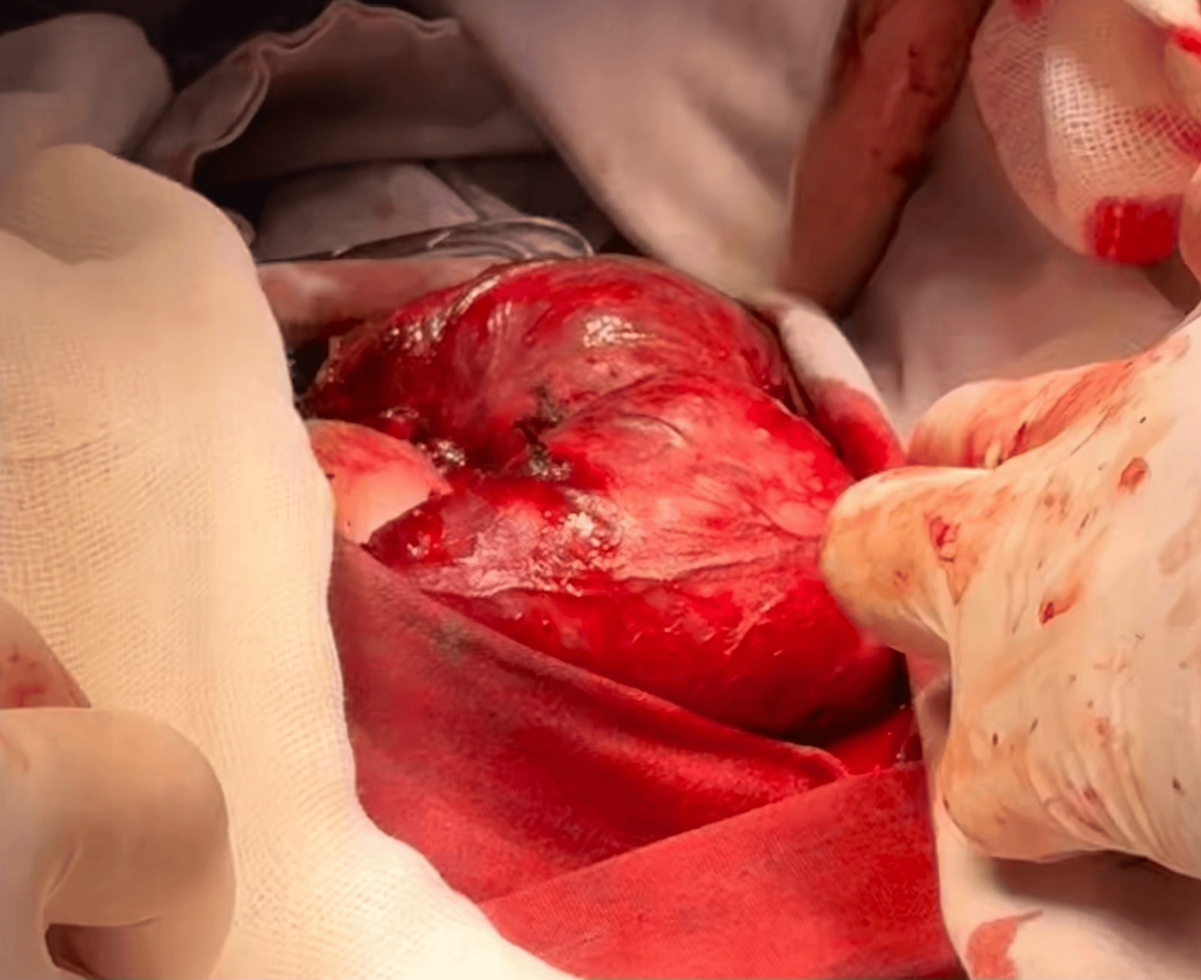
Intraoperative photograph demonstrating the enlarged thyroid gland during surgical exposure.

**Figure 6:**
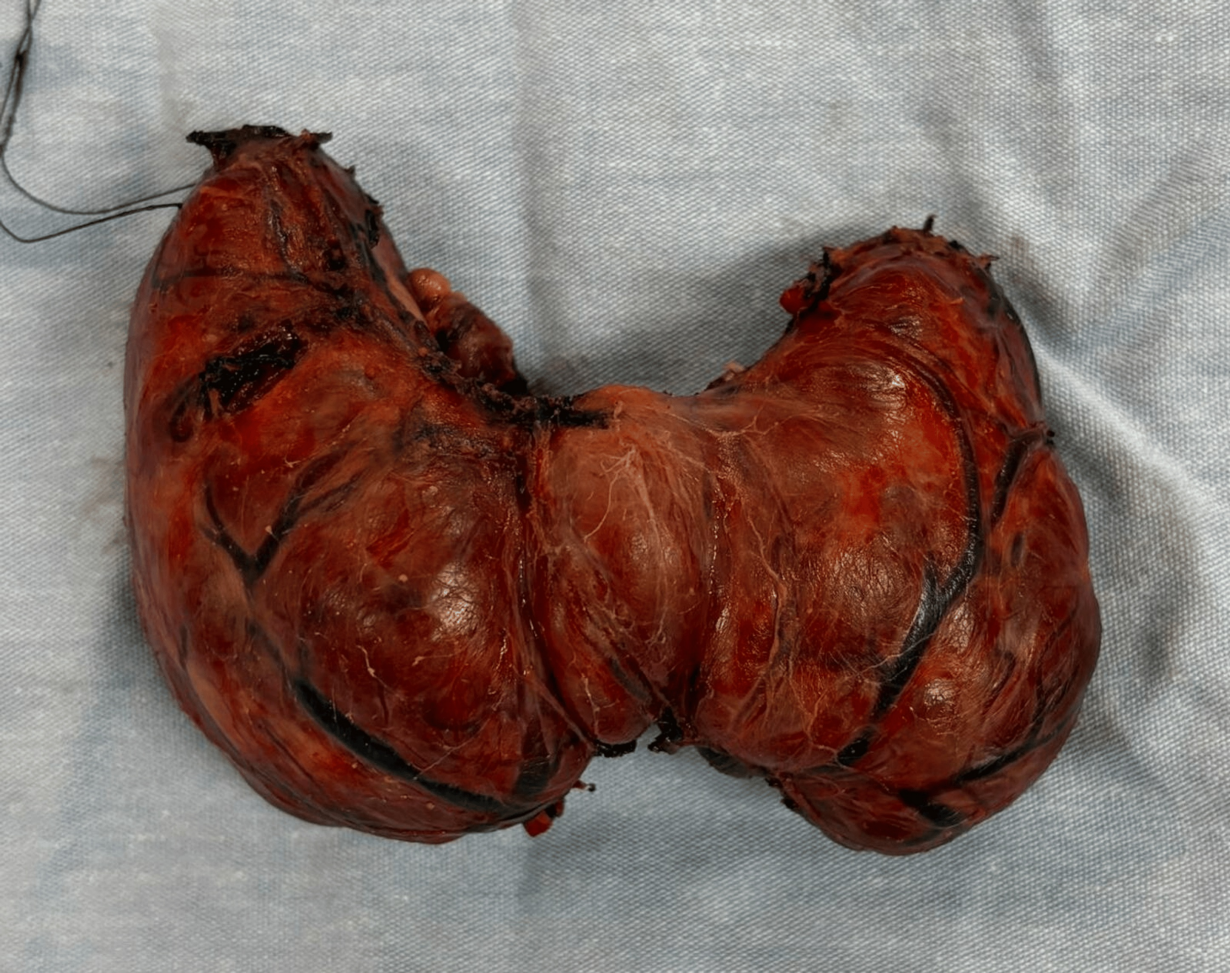
Gross specimen of the thyroid gland following total thyroidectomy demonstrating diffuse bilateral enlargement.

Total thyroidectomy was performed under general anesthesia through a standard Kocher cervical incision. Intraoperative examination revealed a markedly enlarged and highly vascular thyroid gland, with each lobe measuring approximately 8 × 5 × 3 cm (Figure 5). Both recurrent laryngeal nerves were carefully identified and preserved. Adequate vascular control and hemostasis were achieved, a closed suction drain was placed, and the wound was closed in anatomical layers. Gross examination of the surgical specimen demonstrated diffuse bilateral enlargement of the thyroid lobes consistent with hyperplastic goiter (Figure 6).

During the postoperative period, the patient remained under inpatient observation. She experienced mild odynophagia and dysphagia without dysphonia. The surgical drain output was 10 mL of serohematic fluid within the first 24 hours. Serum parathyroid hormone level was 34.6 pg/mL, within normal limits. The patient tolerated oral liquids and subsequently progressed to a solid diet without complications. After 48 hours of observation, the drain was removed, and she was discharged in stable condition.

Histopathological examination revealed a total thyroidectomy specimen weighing 132 g. The right lobe measured 7 × 5 × 4 cm, the left lobe measured 7 × 4 × 4 cm, and the isthmus measured 3 × 2 × 1.5 cm. Microscopic analysis demonstrated diffuse hyperplastic goiter with chronic thyroiditis and no evidence of malignancy.

The patient continued follow-up in the outpatient clinics of general surgery and endocrinology. Treatment with levothyroxine 75 μg daily was initiated, along with insulin glargine 30-0-0 IU and insulin lispro 5-10 IU before meals. 

At follow-up, the patient demonstrated improved glycemic control and stable vital signs. Postoperative laboratory evaluation showed thyroid function tests within the expected range under levothyroxine replacement, with normal serum calcium and parathyroid hormone levels. No recurrence of thyrotoxic symptoms or postoperative complications was observed.

## Discussion

Graves' disease is the most common cause of autoimmune hyperthyroidism and is characterized by thyroid-stimulating antibodies that activate the thyroid-stimulating hormone receptor, leading to excessive production of thyroid hormones and systemic thyrotoxicosis. Although antithyroid drugs remain the first-line therapy in many patients, long-term remission rates are variable, and a considerable proportion of individuals experience persistent or recurrent hyperthyroidism despite prolonged medical treatment. In such cases, definitive therapies, such as radioactive iodine or surgical intervention, become necessary to achieve durable disease control [5].

Recent evidence supports the role of surgical management, particularly total thyroidectomy, as an effective definitive treatment for Graves’ disease. Comparative studies have demonstrated that thyroidectomy provides rapid biochemical normalization and significantly lower recurrence rates compared with prolonged antithyroid drug therapy. A meta-analysis comparing thyroidectomy with antithyroid medications reported higher cure rates and lower recurrence of hyperthyroidism in surgically treated patients, supporting surgery as an appropriate option when medical therapy fails to achieve adequate disease control [6]. In the present case, the patient experienced persistent thyrotoxicosis despite prolonged treatment with methimazole and beta-blockers, fulfilling one of the established indications for surgical management.

Another important indication for thyroidectomy in Graves’ disease is the presence of large goiters or compressive symptoms. Diffuse thyroid enlargement with increased vascularity on Doppler ultrasonography is a characteristic imaging finding in Graves’ disease, often described as the “thyroid inferno” pattern, reflecting increased blood flow within the gland [7]. This diffuse hypervascular enlargement may contribute to compressive manifestations affecting adjacent structures such as the trachea or esophagus. In our patient, imaging studies demonstrated diffuse thyroid enlargement associated with mild tracheal compression, which further supported the decision to pursue definitive surgical treatment.

Severe complications of hyperthyroidism, particularly thyroid storm, represent life-threatening endocrine emergencies associated with significant morbidity and mortality. Patients with a previous episode of thyroid storm are at increased risk of recurrence if hyperthyroidism remains uncontrolled. For this reason, definitive treatment strategies are often recommended in individuals with refractory disease to prevent further decompensation [8]. In the present case, the patient had a prior episode of thyroid storm associated with diabetic ketoacidosis, highlighting the complexity of her endocrine condition and reinforcing the need for definitive management.

The coexistence of type 1 diabetes mellitus further complicated the clinical scenario. Autoimmune thyroid disease frequently occurs in patients with type 1 diabetes as part of autoimmune polyglandular syndromes. Hyperthyroidism can significantly exacerbate metabolic instability by increasing insulin resistance, enhancing hepatic gluconeogenesis, and accelerating intestinal glucose absorption, thereby contributing to poor glycemic control and increasing the risk of metabolic decompensation, particularly in patients with underlying type 1 diabetes mellitus [9].

Total thyroidectomy has become the preferred surgical technique for Graves’ disease in many specialized centers because it removes nearly all thyroid tissue and virtually eliminates the risk of recurrent hyperthyroidism compared with subtotal thyroidectomy [10]. Large clinical series have demonstrated that total thyroidectomy achieves rapid biochemical control and long-term remission with acceptable complication rates when performed by experienced surgeons [11].

Nevertheless, thyroidectomy carries potential risks. The most relevant complications include recurrent laryngeal nerve injury, hypocalcemia secondary to parathyroid gland injury, and postoperative cervical hematoma. Reported rates of transient hypocalcemia range from 4% to 40%, whereas permanent hypoparathyroidism is less frequent but remains a recognized complication [11,12]. Similarly, recurrent laryngeal nerve injury occurs in a small percentage of cases but may significantly affect postoperative voice function [12]. In our patient, both recurrent laryngeal nerves were carefully identified and preserved intraoperatively, and postoperative parathyroid hormone levels remained within normal limits, indicating preserved parathyroid function.

Surgical experience and institutional case volume are also key determinants of postoperative outcomes. Several studies have demonstrated that high-volume thyroid surgeons - typically defined as those performing more than 20-50 thyroidectomies annually - achieve significantly lower complication rates, particularly with respect to recurrent laryngeal nerve injury and permanent hypoparathyroidism [13]. Careful intraoperative identification of critical anatomical structures, therefore, remains essential in minimizing postoperative morbidity.

Adequate preoperative preparation is another critical aspect in patients undergoing thyroidectomy for hyperthyroidism. Optimization with antithyroid drugs and beta-blockers is recommended to achieve euthyroidism whenever possible, thereby reducing the risk of perioperative complications such as thyroid storm [14]. In the present case, prolonged medical therapy had already been implemented before surgery, which likely contributed to the uneventful perioperative course.

Histopathological examination of thyroid tissue in Graves’ disease typically reveals diffuse follicular hyperplasia, papillary infoldings, and varying degrees of lymphocytic infiltration consistent with chronic thyroiditis [15]. These microscopic findings reflect the underlying autoimmune stimulation of the thyroid gland. The pathology observed in our patient demonstrated diffuse hyperplastic goiter with chronic thyroiditis and no evidence of malignancy, findings consistent with those described in the literature [15].

Postoperative management primarily focuses on thyroid hormone replacement and monitoring for complications. Levothyroxine therapy is typically initiated shortly after surgery to maintain euthyroidism and prevent hypothyroid symptoms [16]. In our patient, levothyroxine replacement was started after surgery, and follow-up evaluations demonstrated favorable clinical evolution, with improved metabolic stability and no recurrence of thyrotoxic symptoms.

This case emphasizes that, in patients with refractory Graves’ disease and complex metabolic comorbidities such as type 1 diabetes mellitus, early definitive surgical management should be strongly considered to prevent recurrent life-threatening metabolic decompensation.

## Conclusions

This case highlights the role of total thyroidectomy as a safe and definitive treatment for severe thyrotoxicosis secondary to Graves' disease when medical therapy fails to achieve adequate disease control. In patients with persistent hyperthyroidism, prior episodes of thyroid storm, and complex metabolic comorbidities such as type 1 diabetes mellitus, surgical management was effective in achieving disease control in this case and may be considered as a therapeutic option in similar high-risk clinical scenarios.

This case also underscores the importance of multidisciplinary evaluation in selecting the most appropriate definitive treatment strategy for refractory thyrotoxicosis. When performed in an experienced surgical setting, total thyroidectomy provides rapid biochemical control, definitive resolution of hyperthyroidism, and favorable postoperative outcomes.
